# Protein-Based Nanostructures for Food Applications

**DOI:** 10.3390/gels5010009

**Published:** 2019-02-22

**Authors:** Ana I. Bourbon, Ricardo N. Pereira, Lorenzo M. Pastrana, António A. Vicente, Miguel A. Cerqueira

**Affiliations:** 1 International Iberian Nanotechnology Laboratory, Department of Life Sciences, Av. Mestre José Veiga s/n, 4715-330 Braga, Portugal; ana.bourbon@inl.int (A.I.B.); lorenzo.pastrana@inl.int (L.M.P.); 2 CEB, Centre of Biological Engineering, University of Minho, Campus de Gualtar, 4710-057 Braga, Portugal; rpereira@deb.uminho.pt (R.N.P.); avicente@deb.uminho.pt (A.A.V.)

**Keywords:** nanohydrogel, nanotechnology, bio-based materials, biopolymers, encapsulation

## Abstract

Proteins are receiving significant attention for the production of structures for the encapsulation of active compounds, aimed at their use in food products. Proteins are one of the most used biomaterials in the food industry due to their nutritional value, non-toxicity, biodegradability, and ability to create new textures, in particular, their ability to form gel particles that can go from macro- to nanoscale. This review points out the different techniques to obtain protein-based nanostructures and their use to encapsulate and release bioactive compounds, while also presenting some examples of food grade proteins, the mechanism of formation of the nanostructures, and the behavior under different conditions, such as in the gastrointestinal tract.

## 1. Introduction

In the last decade, there has been an increasing interest in protein-based nanostructures [1,2]. They can be designed to facilitate the encapsulation of diverse classes of bioactive compounds showing interesting characteristics and the potential to be used as a carrier system e.g., they can reach specific targets which are not easily accessed by their counterparts at micro- and macroscale; they can provide new textures and functional properties, being good candidates for intracellular delivery of bioactive compounds; they have a larger surface area-to-volume ratio which is important for in vivo applications; they provide a sizable active compound loading capacity, low density and high dispersion stability in aqueous media; they can be used as a carrier of bioactive compounds with different molecular weights and prolong biological activity in aqueous media. Several types of protein-based structures, such as the cases of hydrogels, solid particles, and emulsions, can be developed depending on the final application (the desired drug release profile, the properties of active compounds such as solubility and particle size).

Some of the most interesting materials that have been used as a base for this development are the hydrogels. Their polymer-based architectures can take up large amounts of water or physiological fluids while maintaining their internal network structure [3,4,5]. The most important functional aspects of hydrogels are related to their swelling and permeability properties. These properties depend much on inter- and intramolecular interactions (e.g., covalent bonds, hydrogen bonding, van der Waals interactions) that can be established, and thus in the way the hydrophilic groups (e.g., hydroxyl and carboxyl groups as well as ethers, amines and sulfates) are organized within the structure. These aspects contribute decisively to important physical hydrogel features such as softness and elasticity [6]. Hydrogels can be classified in different ways, one of the classifications is based on the type of bonds present in the network chains of the gel structure. Polymeric hydrogels are subdivided into two major categories, the physical and chemical cross-linked gels. In physical gels, a heterogeneous organization is formed by molecular entanglements established by hydrophobic associations, ionic interactions, or hydrogen bonding. These gels are also denominated by “reversible” or “pseudo” gels, once they have a restructuring ability. Physical hydrogels are highly influenced by environmental conditions (pH, temperature, ionic strength) which make them sensitive to water (degrade and even disintegrate completely) and temperature (thermo-reversible) [1,7].

Chemical cross-linked gels present higher stability due to the presence of covalent bonds established between different polymer chains. Small molecules (aldehydes) or use of UV light are usually used to cross-link this type of gels. They are stable at different conditions unless the covalent bonds are cleaved [1]. Chemical cross-linked gels have the ability to hydrate and swell until an equilibrium state is reached in response to stimuli [7,8]. 

An interesting strategy to disperse lipophilic compounds into aqueous solution is to prepare oil-in-water (O/W) emulsions. Proteins are also used as emulsifiers, as many of them are surface-active molecules that are used to produce desirable O/W emulsion properties and improve their stability [9]. Proteins have the ability to adsorb droplets to the oil-water interface, lowering the interfacial tension and retarding droplet coalescence by forming a protective membrane around the droplets [10]. Nevertheless, there are some limitations such as the high sensitivity to aggregation at certain pH values (close to their isoelectric point, proteins cannot exhibit strong emulsification properties), at high ionic strengths, and at high temperatures [11].

The functional properties of food proteins in particular, including emulsification, gelation, foaming, and water binding capacity, as well as their applications as ingredients in the food industry have been highlighted during the last few years [5,12,13]. Protein structures are one of the most convenient and widely used matrices in food applications, and in particular protein-based nanostructures have recently attracted a great deal of interest in food engineering and delivery applications [14,15]. In the case of non-solid and semi-solid foods, it is essential to decrease the matrix size to allow its incorporation without affecting food sensory qualities [16]. Furthermore, it is possible to develop new protein vehicles with improved delivery properties by decreasing the structure size from micrometers to nanometers. It is well known that one of the challenges of the food industry is to preserve or increase the bioavailability of bioactive compounds (e.g., vitamins, nutraceuticals and anti-inflammatories) in functional food products aimed at promoting health and well-being. However, the contact with harsh environmental conditions (e.g., gastric fluids, with low pH, high concentrations of salts and ionic strength), and the often low solubility during gastro intestinal digestion results in only a small proportion of bioactive molecules remaining available for oral administration, which limits the activity and potential health benefits of bioactive compounds [17]. The production of systems which are able to maintain the activity of these molecules until the time of consumption and physiological delivery is being extensively investigated by food technologists. Protein-based delivery systems hold promise in providing such protection to bioactive molecules and complying with the requirement of the use of the compounds; generally Recognized as Safe (GRAS), which is mandatory in the food industry [18].

Based on the knowledge of protein physicochemical properties, this review describes the potential role of food proteins to produce nanosized structures in order to create new delivery systems, and addresses their potential applications within the food industry.

## 2. Proteins and Their Functionality

Proteins are widely applied in the food industry due to their ability to add unique functional properties such as emulsifying, foaming, gelling, and solubility attributes [19] (Table 1). The functional properties of proteins have a high impact in final food products, namely in organoleptic (color, flavor, odor), kinesthetic (mouthfeel, texture, smoothness), and textural (elasticity, cohesiveness, chewiness, adhesiveness) properties. 

The selection of a suitable protein or combination of proteins to produce protein-based systems is based on their properties which depend on both intrinsic (e.g., molecular structure, composition, solubility) and extrinsic factors (e.g., temperature, chemical environment, pH) [20]. The type, number, and particular sequence of amino acids in a protein determine the molecular weight, conformation, electrical charge, hydrophobicity, physical interactions, and other functionalities. Food proteins may serve as an effective carrier of bioactive molecules in formulated foods because of their ligand binding properties and high nutritional value. However, an important handicap of the use of proteins is their allergenicity. There are some proteins such as milk, soy, or egg proteins that may cause an allergic or intolerance reaction in some consumers [21]. Some of the most important food proteins characteristics are discussed below.

### 2.1. Milk Proteins

Milk proteins are widely available, inexpensive, natural and GRAS materials with numerous functional properties, being a solution, as a vehicle to encapsulate and control the release of bioactive compounds in the food industry [22,23]. In milk, there are two major protein types: whey proteins and caseins [24]. Whey protein-based matrices are being widely used in food products not only for their high nutritional and biological value (digestibility, amino acid pattern, high biological value and sensory characteristics) but also for their ability to form cold- and heat-set hydrogels [25,26,27].

Caseins are phosphoproteins precipitated from milk with a pH around 4.6, comprising different types of milk proteins, namely α-, β- and κ-caseins, correspond to approximately 80% of the total protein in milk. Caseins can be found in association colloids which are denominated as casein micelles, being an excellent example of their application as a vehicle of bioactive compounds [22]. 

The following proteins belong to the whey protein group in higher quantities: β-lactoglobulin, α-lactalbumin, and in lesser amount serum albumin, lactoferrin, immunoglobulins, and protease peptones. Whey proteins are globular proteins, corresponding to 20% of the total protein present in milk, being more heat sensitive, and less sensitive to calcium than caseins [28].

β-lactoglobulin (β-Lg) is a globular protein with a molecular weight of 18 kDa and is very stable in acidic conditions [29]. It is generally in a dimer form at the isoelectric pH of 5.2 and alkaline pH range. The three-dimensional structure of this protein is greatly affected by temperature. At temperatures above 60 °C it starts to be denaturated also resulting in the appearance of some monomers which are responsible for milk intolerance in humans [29]. 

α-Lactalbumin (α-La) is a globular protein with ellipsoid shape and a molecular weight around 14 kDa. This protein is thermostable, due to the presence of four disulfide bonds. α-La has different bioactive properties such as the ability to bind to calcium and antioxidant activity [24]. Bovine serum albumin (BSA) is a globular protein having a molecular weight of 66 kDa, 580 amino acid residues and 17 intra-chain disulfide bonds. BSA contains three domains specified for metal-ion binding, lipid binding, and nucleotide binding [30]. Glycomacropeptide (GMP) is a sialylated phosphorylated peptide with an isoelectric point between 4 and 5. GMP is a C-terminal part of kappa-casein which is released in whey during cheese making by the action of chymosin. GMP is a biologically active component with unique chemical and functional properties such as antibacterial activity, the modulation of immune system responses and the regulation of blood circulation [31]. Some of these bioactive properties of GMP are attributed to its carbohydrate moieties attached to the peptide.

Lysozyme is a protein secreted in milk and its molecular weight is around 15 kDa. Lysozyme is used for food applications due to its antimicrobial activity, being active against Gram-positive bacteria [32].

Lactoferrin is a basic, positively charged iron-binding glycoprotein of the transferrin family with a molecular weight of 80 kDa and an isoelectric point around 8.0–8.5, present in various external secretions of mammals such as milk [33,34]. One of the main interests in lactoferrin resides in its various biological activities, such as antimicrobial, anti-inflammatory, antitumor, immuno-modulatory, and enzymatic activities.

### 2.2. Soy Protein

Soy proteins are abundant, renewable, and biodegradable, thus being attractive for application in food products [35]. They are mainly globular and have a molecular weight ranging from 8 to 600 kDa with an isoelectric point between 8.0 and 8.5 [36]. Soy proteins can be classified into four categories—i.e., 2S, 7S, 11S, and 15S fractions—according to their sedimentation process. The fractions 7S (conglycinin) and 11S (glycinin) are the major fractions in soy protein structure (35% and 52%, respectively) and are related to the protein functionality. Soy proteins are also used to a great extent to produce food gels, in particular the purified rich fractions of glycinin and β-conglycinin (main proteins in soybeans) and soy protein isolate (mixture of soy proteins). Heat denaturation is a prerequisite for gel formation independently of pH and ionic strength conditions. Soy β-conglycinin gels can be formed at temperatures of about 55–70 °C, while glycinin gels form at about 70–95 °C. For example, tofu, probably the most famous soybean gel, is produced by heating soybean milk followed by addition of salts (e.g., Ca^2+^ or Mg^2+^) or acidification (using glucono-δlactone), which promotes protein aggregation by neutralizing the negatively charged groups of denatured proteins.

### 2.3. Other Proteins

Ovalbumin (OVA) is the predominant protein fraction in egg white and is a highly functional food protein that is frequently used in food matrices’ design. It has a molecular weight of 47 kDa and an isoelectric point of 4.8 [32]. OVA has more than 50% hydrophobic amino acids, used to stabilize emulsions or foams. Moreover, this protein has also been used to form gel networks and encapsulate bioactive compounds [32]. Denaturation of OVA determines the optimum temperature for gel formation and contributes to the increase of gel strength when the temperature is raised above 80 °C [33].

Collagen is the structural building material of vertebrates and the most abundant mammalian protein that accounts for 20%–30% of total body proteins. Collagen has a unique structure, size, and amino acid sequence which results in the formation of triple helix fiber. Collagen is an abundant, renewable, and biodegradable protein, making it attractive to be applied in food products [34]. 

Gelatin is obtained by controlled the hydrolysis of fibrous, insoluble collagen which is widely found as the major component of skin, bones, and connective tissue [35]. Gelatin is considered an interesting biodegradable material, non-toxic and inexpensive and has, therefore, an immense potential to be used for the preparation of colloidal drug delivery systems [36]. This protein is widely used in the food industry as it has the ability to form reversible thermosetting gels (resembling many polysaccharides) with unique rheological properties that melt at 35–37 °C (typical melt-in-the-mouth-texture). Gelatin protein molecules swell in cold or hot water adsorbing up to ten times their weight, and when heated to temperatures above the melting point, give rise to a viscous solution of random-coiled linear polypeptide chains. Upon subsequent cooling to temperatures of 20 °C, a gelatin gel is produced due to the formation of a network of polymeric strands stabilized by hydrogen bonding interactions [37].

Zein is a protein present in corn with a molecular weight ranging between 22 and 27 kDa and an isoelectric point of 6.2 with high solubility in aqueous alcohol solutions [36,37]. This protein has a high proportion of nonpolar amino acids which are responsible for the solubility behavior of zein [38]. This water-insoluble protein is classified as GRAS by the US Food and Drug Administration and is widely used in food [39]. This biodegradable and biocompatible protein has been used as a vehicle for the delivery of active compounds (e.g., enzymes, drugs, and essential oils) [39,40,41,42,43].

## 3. Methodologies to Fabricate Protein-Based Nanostructures

Protein-based nanostructures, such as particles, gels, and fibers, can be produced using different methodologies as described in the next sections.

### 3.1. Gelation Mechanisms

#### 3.1.1. Denaturation of Globular Proteins

Gelation of proteins usually requires a driving force to unfold the native protein structure, followed by an aggregation process to give a three-dimensional network. Protein–protein interactions, aggregations mechanisms, and types of protein aggregates, as well as processing techniques, determine much of the produced structures. Protein aggregates serve as “building blocks” to the development of food-grade nano- and micro-network gels. Without a heating step, the protein network structures formed, barely remain stable in water [44]. At denaturation temperature globular proteins start to unfold, and depending on the balance of attractive and repulsive interactions between them, they can remain as individual denatured molecules or form fibrillar or particulate aggregates [44]. These outcomes are extremely dependent both on the heating method (direct and indirect) and the heating conditions, such as temperature, heating rate, treatment time, and environmental conditions (e.g., pH, ionic strength, and salt addition). However, aggregation mechanisms, as well as types of protein aggregates, are defined by the chemical environment of the aqueous protein solution, namely: pH, ionic strength, and protein concentration. In this sense, physical and chemical methods can be used as a driving force for the development of protein systems, and generally involve the use of heat, pressure, and enzymatic reactions among others. Recently, the use of emergent electroheating technologies (such as ohmic heating) have also been associated with protein structural disturbances that can change their aggregation pathways at the nano-scale [45,46]. A comprehensive understanding of all these factors involved in protein denaturation will allow a better design of nanostructures with the intended functionalities. 

##### Thermally-Induced Gelation

One of the most common methods for the formation of gels, from macro- to nanoscale, with globular proteins is the application of heat [46]. Temperature affects the thermodynamic stability of whey proteins by reducing the activation energy and increasing the frequency of molecular collisions and the enhancement of hydrophobic interaction, which is a crucial step for the occurrence of physical protein aggregation. Consequently, high temperature, above the protein denaturation level is a key parameter for accelerating whey protein aggregation [26]. Generally, globular proteins, such as β-lg, aggregate spontaneously and irreversibly if they are denatured at temperatures above 60 °C [47]. Above the denaturation temperatures thermal gelation and hydrogel production can be seen as a simple sequential three-step process, which involves: (1) unfolding of native protein and exposition of hydrophobic amino acid residues, (2) primary and secondary aggregation of unfolded proteins, where covalent and non-covalent bonds are established and (3) development of a protein network structure, when the aggregation exceeds a critical point of the concentration. Above a critical gel protein concentration, a system spanning network is formed which is tight and stable, but at lower concentrations it collapses under gravity, leading to precipitation or the formation of heterogeneous systems. These are some of the key concepts of the general model for globular protein gelation proposed by Ferry [48]. This model describes gelation based on the denaturation temperature, a critical protein concentration, a critical gelation time that is dependent on the rate of denaturation, and aggregation. More recently, some complexity has been added to this model taking into account the reverse pathway of protein folding and formation of intermediate states of protein aggregates that can form disordered aggregates or amyloid fibrils [49]. In this sense, it is important to clarify the molecular approach of protein aggregation, thus the precise details of association mechanisms that strongly impact the rheological performance of gels in food materials should be fully explained [50]. This technique has been widely used to obtain protein nanoparticles for food applications [51,52,53].

##### Acid-Induced Gelation

Acid-induced gelation occurs by lowering the pH (normally to that corresponding to the pI) and thus changing the net charge of protein molecules as well as the interactions between protein and solvent. When the protein has a zero net charge, repulsive forces are minimized and aggregation occurs. The mechanisms of acid-induced gel formation can be explained by the fractal aggregation theory. Fractal aggregation assumes that spherical particles of the radius can move by Brownian motion and that they can aggregate when they encounter each other. These aggregates formed can then aggregate with each other giving rise to fractal aggregates or clusters, which are considered to be the building block of the gel [54]. However, acid gelation often requires an initial physical treatment (heating or high pressure) in order to unfold the native structure of the protein molecule, exposing it to further reactions. Andoyo et al. showed the possibility of producing different sizes of microbeads using whey proteins with acid gelation, but at nanoscale these kinds of examples are scarce [55].

##### Ionic Gelation

Increasing the ionic strength in protein solutions through the addition of salt ions can screen or shield the electrostatic charges on the protein molecules or aggregates. As a consequence, electrostatic repulsive forces between the molecules are minimized and gelation occurs. When the pH of the protein solutions is well above the pI of the proteins, divalent ions can act as an intermolecular crosslinking agent by promoting the bridging of the proteins. Ionic-induced gelation is often called cold gelation [56]. In contrast with heat-induced gelation, cold gelation occurs below denaturation protein temperatures. However, it requires a pre-denaturation step (by heat, pressure or enzymatic treatment) in which proteins are unfolded, losing their native state and giving rise to the appearance of soluble aggregates. This denaturation pre-treatment is then followed by salt addition, which results in the formation of a network mediated via interactions between cationic agents and protein soluble aggregates at environment temperatures [56]. Depending on the protein:cationic agent ratios different gel networks structures can be obtained. Cold-set protein hydrogels can be obtained by adding cationic agents such as ferrous, calcium or barium salts to solutions of denatured globular proteins [57,58].

##### Enzymatic Gelation

A way to control the aggregation/cluster state of proteins that is complementary to heat-induced gelation is the use of certain enzymes that can induce artificial covalent cross-links into food proteins. The best-known enzyme, available for use in the food industry, is microbial transglutaminase (TG: EC 2.3.2.13), that catalyzes the formation of cross-links between lysine and glutamine residues [59]. Milk caseins are particularly good substrates for TG-catalyzed cross-linking. Equally, whey proteins, when partly unfolded, can be polymerized to a great extent using TG [60]. Depending on the enzyme concentration, the incubation time, and the type and concentration of proteins available, the functional benefits of TG-treatment are the gelation of proteins, the increase of gel strength and elasticity, and the higher water-holding capacity [61]. Other classes of protein cross-linking enzymes are oxidative enzymes such as peroxidase (POD; EC 1.4.3.13) and polyphenol oxidase (PPO; EC 1.14.18.1). Horseradish peroxidase can induce cross-linking of ovalbumin, β-lg, and BSA, yielding various oligomers and polymers in the presence of hydrogen peroxide and a low molecular weight hydrogen donor. PPO is an enzyme that can be used to cross-link casein in the presence of a low molecular weight phenolic compound such as caffeic acid. Overall, food functional effects of enzymatic protein cross-linking also include improvements of gelation properties and emulsion stability as well as enhanced heat-stability [62,63,64].

### 3.2. Other Methods and Materials to Fabricate Protein-Based Nanostructures

Protein-based nanostructures can also be produced in combination with other polymers or other biopolymers. These mixtures can be used to improve the stability, or change the hydrophobicity level or density as well as the synergistic interactions between different proteins or between proteins and polysaccharides. Different delivery systems (that combine different biopolymers) have been developed and are presented in Table 2.

A mixture of two biopolymers can form one-phase or two-phase systems, depending on different factors: (1) the type of biopolymer, (2) the ratio between biopolymers in the solution and (3) the environmental conditions. When two biopolymers are distributed throughout the system as soluble complexes or individual molecules, a one-phase system is formed. However, if the solution separates into two distinct phases composed of different concentrations of the biopolymers, a two-phase system is formed instead [69]. Phase separation can occur through two types of mechanisms which are presented below. However, in recent years, some new methodologies have appeared as a way to produce different types of protein-based nanostructures (e.g., particles and fibers) such as nanospraydrier and electrospray/electrospinning.

#### 3.2.1. Associative Separation

In a two-phase system a strong attraction occurs between two biopolymers, forming one phase rich in one polyelectrolyte and other phase being depleted in both polyelectrolytes. The phase which contains the biopolymers can be a precipitate or a complex coacervate, depending on the charge of the biopolymer and the strength of the attraction between the biopolymers. 

Complex coacervation occurs when an electrostatic attraction is established between molecules (e.g., proteins, polysaccharides or between proteins and polysaccharides). These systems are extremely sensitive to environmental conditions (pH, temperature, and ionic strength) as well as to the total polyelectrolyte concentration and the protein/polyelectrolyte ratio. Complex coacervation is considered a simple method of creating particles that can be used as a carrier of active compounds. Nevertheless, there are several factors that currently limit their application in the food industry. The coacervate phase has low stability over a relatively narrow range of pH values, and the complex can disintegrate (when the molecules have strong similar charges) or form precipitates (when the molecules have strong opposing charges). These structures, namely particles, are considered unstable when the ionic strength increases, which limits their application in some food products as they tend to coalesce over time, leading to a gradual increase in the mean particle size and eventually to macroscopic phase separation [2]. This technique is usually reported to produce nano-coacervates for encapsulation of bioactive materials [15,70,71].

#### 3.2.2. Segregative Separation

In segregative separation there is a repulsion between the two different biopolymers. This type of phase separation is usual when the biopolymers are uncharged or when the two biopolymers have similar charges. At low biopolymer concentration, the two biopolymers are mixed and can form a one-phase system, however, when the biopolymer concentration exceeds a certain level, phase separation occurs and a two-phase solution is formed with each phase being rich in one type of biopolymer and depleted in the other. This mechanism produces microstructures such as “water-in-water” emulsions or “oil-in-water” emulsions. These systems are considered kinetically stable and can result in novel microstructures with new functional and rheological properties for the food industry [69].

#### 3.2.3. Physical Self-Assembly of Interactive Polymers

Protein-based structures can be formulated by the self-assembly method with charged polymers, where interactions between the biopolymers occur for example by non-covalent interactions (e.g., van der Waals interactions and hydrogen bonding). Different protein-based particles can be prepared based on this methodology. For example, Yu et al. (2006) prepared nanogels by self-assembly of two proteins with opposite charge (ovalbumin and lysozyme) and by thermally-induced gelation [72]. The utilization of this methodology using biopolymers of opposite charge, where the size is controlled by polymer concentrations and different environmental conditions (e.g., pH, temperature, and ionic strength), is a strategy for developing nano-hydrogels. Bourbon et al. (2015) applied this technique to develop complex coacervates between lactoferrin and glycomacropeptide and applied thermal gelation on these complexes to obtain stable nanohydrogels [65].

#### 3.2.4. Water-in-Oil Heterogeneous Gelation

Water-in-oil (W/O) heterogeneous gelation involves the emulsification step of aqueous droplets of the protein (gelling agent) stably dispersed in a continuous organic phase with the aid of oil-soluble surfactants [73]. This method is used to better control the size of particles. The proteins within the water droplets of the W/O emulsion are then cross-linked using enzymes [74], thermal denaturation [75] UV-light induced cross-linking [76], or addition of cross-linking ions [77]. The general methods for W/O heterogeneous gelation include inverse (mini)emulsion, the reverse micellar method, and membrane emulsification, and a brief explanation is presented below [78].

The inverse emulsion method yields kinetically stable W/O macroemulsions at, below, or around the critical micellar concentration of surfactants [79]. The protein in the aqueous droplets is then chemically and physically crosslinked with the appropriate crosslinking agents, producing submicron-sized gels.

The reverse micellar method can be used to prepare protein-based microgels. The reverse microemulsion method requires the addition of a large amount of oil-soluble surfactants above the critical threshold, producing thermodynamically stable micellar solutions.

In the membrane emulsification technique, the to-be-dispersed phase is passed through a membrane with controlled pore size. Under controlled conditions, hydrogel particles with specific morphology are formed on the surface of the membrane, these hydrogels being subsequently recovered by a continuous phase that flows across the membrane.

#### 3.2.5. Micromolding, Photolithography, and Microfluidic Preparation

These methods have been used to prepare biopolymer particles such as nanoparticle gels. In the micromolding method, a biopolymer solution is placed onto a mold and induced to form a gel by controlled factors (e.g., temperature or gelling agents). After this process, particles are removed from the mold and used. The microfluidic method allows for the preparation of various biopolymer-based particles upon the design of various microfluidic channels. These methods allow the preparation of particles with sizes below 1 μm, with different shapes for specific delivery applications. To prepare particles by these methods the development of specific molds, photolithographic molds, and microfluidics devices is necessary. Furthermore, economic challenges have limited large scale production by these techniques [80].

#### 3.2.6. Spray-Drying

**Figure 1:**
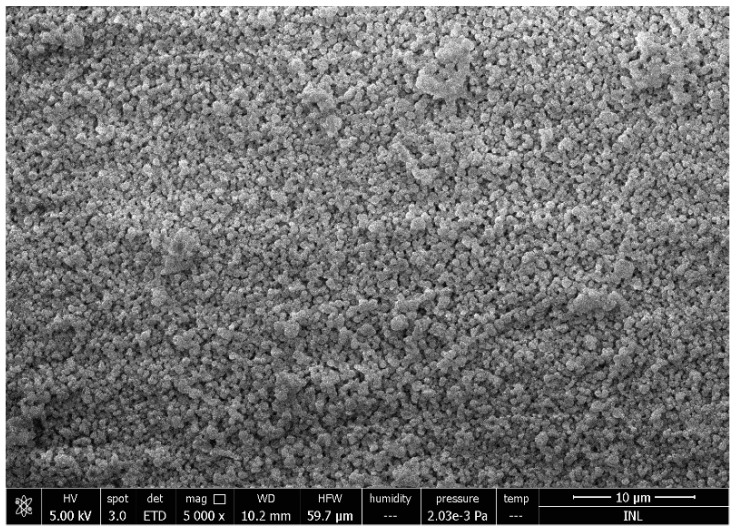
Lactoferrin particles at the submicron scale produced by a nanospraydrier. The image was obtained by scanning electron microscopy.

Spray drying is a well-established method commonly used in the pharmaceutical and food industries for the production of a dry powder from a liquid phase. This technique allows the manipulation of various particle properties such as particle size, bulk density, and flow properties by the adaption of the process parameters or spray dryer configuration (e.g., flow rate, drying temperature, and the separation of the dried material from the air). During the spray drying of nanoparticles, a biopolymer solution feedstock is atomized into a spray of fine droplets and then brought into contact with the hot drying air at a sufficient temperature for moisture evaporation to take place. As the moisture evaporates from the droplets, a solid product (powder) is formed and recovered from the drying air [63]. This has the advantage of forming a convenient dry powder product with extended shelf life and low storage or transport costs [64]. Recently, a new generation of laboratory scale spray dryers that is able to produce particles at nano scale, has been introduced. The Büchi Nano Spray Dryer B-90 relies on its vibration mesh spray technology, creating tiny droplets (before evaporation) in a size range of a smaller order of magnitude than classical spray dryers. In this equipment, the electrostatic particle collector allows the efficient separation of the dried particles. Some work has been done to prepare protein particles to encapsulate active compounds (vitamins, antioxidants, metals [63]). Figure 1 shows the morphology of lactoferrin-based particles produced by a nanospraydrier.

#### 3.2.7. Electrospray/Electrospinning

**Figure 2:**
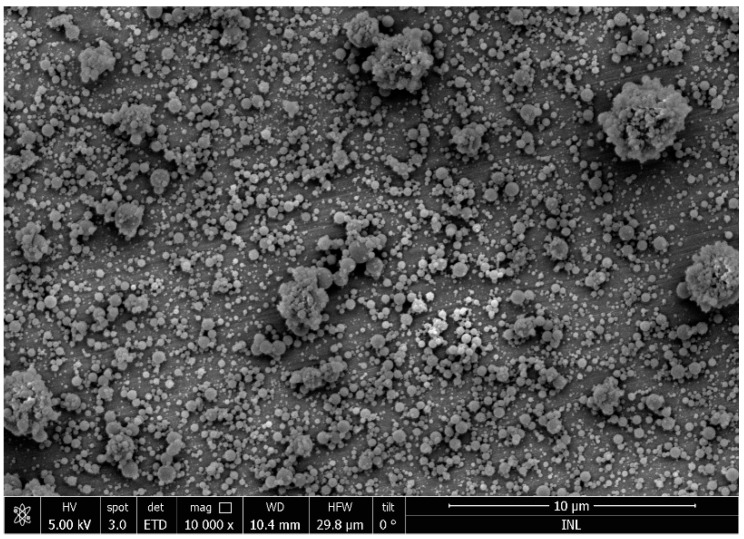
Lactoferrin particles at the submicron scale produced by electrospray. The image was obtained by scanning electron microscopy.

Electrospray/electrospinning is a relatively new technique for the development of biopolymer nanoparticles and nanofibers [81]. A high voltage is applied in a biopolymer solution which emits a liquid jet stream through a nozzle, forming controlled liquid droplets. Bioactive compounds can be easily incorporated into the nanostructure with high efficacy using this method. Electrospraying has emerged as a similar technique to electrospinning, which uses an analogous technology for the production of nanostructures. This technique has numerous advantages such as scalable synthesis, reproducibility, no need of heat, and high encapsulation efficiency. Furthermore, the nanoparticles produced by electrospraying/electrospinning are considered stable and without any loss of their bioactivity [82,83,84]. Figure 2 shows the morphology of lactoferrin-based particles produced by electrospray.

## 4. Protein-Based Nanostructures: A Vehicle to Transport Bioactive Compounds

Protein-based nanostructures are one of the most used systems to encapsulate different types of bioactive compounds (hydrophilic or lipophilic compounds with different molecular weight, solubility, etc.) [5]. These nano-systems present a high surface area which increases the possibility to uptake active compounds and improve their absorption and bioavailability [85]. Numerous works have been published indicating the influence of environmental conditions on protein-based nanostructures and evaluating the release profile of active compounds encapsulated in this type of system. An improvement of bioavailability and a controlled release of encapsulated compounds were observed when protein-based nanostructures were employed, which is an important step to create new solutions for delivery of bioactive compounds at a specific target. In addition, protein-based nanostructures allow some drawbacks related with other nanostructures to be overcome such as the production protocol and the low loading capacity. They can be produced in an isolated way (i.e., without the interference of the bioactive compounds) and designed to spontaneously load bioactive molecules through electrostatic, van der Waals, and/or hydrophobic interactions between the bioactive compounds and the polymer matrix, during the gel folding process. All these aspects contribute to the formation of stable nanostructures. Moreover, the ability of these nanostructures to produce a response (e.g., swelling) to environmental stimuli (e.g., temperature, pH, ionic strength, or enzymatic conditions) makes them very interesting systems to deliver bioactive compounds locally to specific sites and at a particular time in the GI tract, which allows the inherent instability of this complex route and the low permeability of the intestinal mucosa to be overcome [86]. On the other hand, these structures may present limitations, particularly if produced by physical gelation, as the nanohydrogels contain labile bonds in the polymer networks which are susceptible to being disrupted under physiological conditions in the GI tract [34]. Protein-based nanostructures can be prepared from several proteins (whey proteins, zein, collagen) with different techniques, the gelation process being the most commonly used method. It is possible to find in the literature different works reporting the ability of protein nanohydrogels to incorporate and release hydrophilic and lipophilic bioactive compounds such as drugs, unsaturated fatty acids, vitamins, as well as peptides—see Table 3.

Depending on the nature of the bioactive compounds incorporated in the nanostructure, it is possible to obtain different release mechanisms during the digestion process. Hydrophilic compounds are released from a protein matrix by diffusion, whereas lipophilic compounds are released mainly by enzymatic degradation of the protein matrix in the GI tract [34]. Table 3 presents some examples of bioactive compounds efficiently incorporated into protein-based nanostructures, with information regarding the encapsulation techniques and their main limitations. The delivery of bioactive compounds at a specific target (e.g., mouth, stomach, small intestine, and colon) with the bioactive properties is still a challenge. The design and manufacture of protein-based systems are extremely important in order to resist severe environmental conditions (e.g., resistant to gastric fluids, when the nanostructure is designed to deliver a bioactive compound in the colon). For instance, nanostructures composed of peptides or proteins have a high level of GI degradation by digestive enzymes [93].

## 5. Controlled Release

The controlled release of functional compounds from biopolymer-based structures at the macroscale has been extensively studied. Most of these studies were performed in drug delivery systems. In foods, the main applications of controlled release from biopolymer-based structures are related to the controlled release in the human gastrointestinal environment, where the development of particles to deliver nutrients in the intestine has been explored [94]. The controlled release of active compounds has numerous advantages in food products such as: prolonging the bioactivity through time (e.g., from vitamins and antioxidants) and protecting sensitive bioactive compounds from enzymatic or acidic degradation in the gastrointestinal tract [34]. It is extremely important to understand the transport properties of compounds in a matrix and how are they influenced by the size of the system (macro-, micro- or nanoscale), as this provides valuable information regarding their behavior e.g., throughout the food products’ shelf-life. The release of bioactive compounds from bio-polymeric matrices may occur due to different mechanisms, such as Fick’s diffusion, the relaxation of the matrix which is dependent on matrix swelling and erosion. Once the system has come in contact with different environmental conditions (e.g., pH, temperature, ionic strength), a different mechanism may prevail. These mechanisms may be generally classified as belonging to three different types: ideal Fickian diffusion (Brownian transport), Case II transport (polymer relaxation-driven), and anomalous behavior (ranging from Fickian diffusion to Case II transport) [33].

In recent years, several works concerning the release of functional compounds from microparticles have been reported [95,96,97]. Comparatively, few works can be found on release mechanisms involved at the nanoscale. Literature suggests that in the case of biopolymer particles, the release behavior depends mostly on the permeability and on the disassembly or erosion of the structure, together with other experimental variables which are much less reported, as well as on the dependence on the matrix composition and the way the compound is released—i.e. release may follow a Fickian or an anomalous transport behavior [98]. Also, some authors attribute the observed behavior to a Fickian transport through the particle, followed by release due to polymer dissolution. However, there are still a lot of unknown variables affecting the release of compounds from protein-based nanostructures, especially regarding the effect of environmental conditions on the matrix of the protein and its structure. During gastro intestinal digestion, proteins are highly degraded (by low pH, high ionic strength, and hydrolysis by enzymes) and consequently, their bioaccessibility is low. One of the strategies to improve the protein-based structure stability during gastro intestinal digestion is the application of a biopolymer layer to protect the protein particles. Recently, Bourbon et al. (2018) evaluated the effect of gastro intestinal conditions on protein nanohydrogels, with and without a chitosan layer, on active compounds release (curcumin and caffeine). These authors concluded that the chitosan layer improved the stability and bioaccessibility of active compounds during in vitro digestion. Furthermore, the degradation of the lipophilic compound was lower when compared with the system without the chitosan layer [99].

## 6. Conclusions

The increasing number of publications dealing with proteins for the production of nanostructures shows the strong interest of food scientists in this area, and which can open new horizons for interesting applications by the food industry. Their use for the encapsulation and controlled release of bioactive compounds seems to be one of the most interesting applications. One of the challenges to be overcome is finding technological solutions to scale-up their production in a competitive way. Another issue to address is concerned with more comprehensive knowledge on the interaction of the produced structures with the human body, and in this case, toxicological studies should be performed in order to confirm their safety is guaranteed.

## Figures and Tables

**Table 1:** Some examples of food proteins with the ability to form nanostructures and their related functional properties.

Proteins	Functional Properties
β-lactoglobulin	Emulsifying, foaming and gelling properties
Bovine serum albumin	Foaming, and emulsifying properties
α-Lactalbumin	Gelling properties and fat and flavor binding
Casein	Emulsifying, foaming and gelling properties,
Lactoferrin	Gelling properties
Gelatin	Emulsifying, gelling properties
Soy protein	Gelling properties and thermal stability
Wheat proteins	
Corn zein	

**Table 2:** Micro- and nanostructures produced by the combination of proteins and other biopolymers.

System	Biopolymers	Production Techniques	References
Nano-hydrogels	Lactoferrin and Glycomacropeptide	Thermal gelation	[65]
Particles	β-lactoglobulin and pectin	Thermal gelation	[66]
Coacervates	Lactotransferrin and β-lactoglobulin	Coacervation	[15]
Conjugates	Weight protein isolate and pectin	Coacervation	[67]
Hydrogels	β-lactoglobulin and chitosan	Thermal gelation	[4]
Supramolecular structures	α-lactalbumin and glycomacropeptide	Self-assembly	[68]

**Table 3:** Selected bioactive compound-loaded nanohydrogels for food/oral applications.

Biopolymer	Bioactive Compounds	Nanoencapsulation Techniques	Application	Limitations	Reference
β-lactoglobulin	Curcumin	Complex formation	Food based nanocomplex	Environmental conditions (temperature, pH, ionic strength)	[87]
β-lactoglobulin and pectin	ω-3 polyunsaturated fatty acids	Electrostatic nanocomplexes	Clear acid drinks	Heat stability	[88]
Lysozyme and Sodium Carboxymethyl Cellulose	5-fluorouracil	Polyelectrolyte complex coacervation	Bioactive compound carrier	Burst release of the active compound	[89]
β-lactoglobulin and hen egg white protein	α-tocopherol	Salt-induced gelation of the proteins	Bioactive compound carrier	n.a	[14]
Lactoferrin and Glycomacropeptide nanohydrogels	Curcumin and caffeine	Thermal gelation	Bioactive compound carrier	Heat stability	[33]
Whey protein isolate	Anthocyanin rich extracts	Coacervation	Encapsulate and protect anthocyanin	Thermal degradation	[90]
Whey proteinconcentrate	Folic Acid	Electrospray particles	Encapsulation of bioactive compounds	n.a	[91]
Gelatin	Polyphenols	Electrospray particles		n.a	[92]
